# Innovation of the Education of College Students' Outlook on Life Following Positive Psychology Under the Theory of Educational Psychology

**DOI:** 10.3389/fpsyg.2021.739284

**Published:** 2022-01-04

**Authors:** Xiao Long, Peiyao Chen, Qingquan Liu, Fengrui Zhang, Chao Lu

**Affiliations:** ^1^School of Foreign Studies, Hunan University of Science and Technology, Xiangtan, China; ^2^School of History and Culture, University of Birmingham, Birmingham, United Kingdom; ^3^College of Entrepreneurship, Jiaxing University, Jiaxing, China; ^4^Trinity College, University of Cambridge, Cambridge, United Kingdom; ^5^Faculty of Education, East China Normal University, Shanghai, China

**Keywords:** educational psychology, positive psychology, educational innovation, college students' outlook on life, educational theory

## Abstract

The study expects to find a better way to improve the teaching quality of the education of college students' outlook on life, based on the theory of educational psychology. First, the relevant theories of positive psychology are introduced and expounded, and the importance of the education of college students' outlook on life is analyzed. Second, the current situations of college students' outlook on life and the education of their outlook on life are investigated through a questionnaire survey, and the problems presented in the questionnaire are analyzed. Then, the correlation between positive psychology and the education of college students' outlook on life is explored, and their mutual connection is analyzed. The results are as follows: 77.4% of the college students have periodical aims and work hard for them; 80.8% of the students think that the realization of life goals rely on hard work, accounting for the largest proportion; when they encounter setbacks, more than 80% of the students choose to work hard to overcome them; 69.2% students think that their outlook on life comes from self-learning and exploration. According to college students' outlook on life in China and other countries, there are many problems in the education of college students' outlook on life, and the teaching quality of the education of college students' outlook on life is backward. The combination of positive psychology and college students' education of college students' outlook on life under the theory of educational psychology provides new ideas and ways for college students' education of college students' outlook on life. The conclusion of this study promotes the innovation of the education of college students' outlook on life.

## Introduction

College students are the main force of China's future development, and their outlooks on life are formed during this important period. Faced with a large amount of information, college students tend to be affected by that information, which gives them trouble in establishing a correct outlook on life. Therefore, colleges and universities must strengthen the education of college students' outlook on life. Sharaievska and Harmon (2021) concluded that life quality can be guaranteed only when correct ideas are formed by investigation and research on college students.

Ideological and political courses in colleges and universities are an important part of the education of college students' outlook on life. They help students develop a healthy and positive attitude and establish a correct outlook on life. However, there are still many problems in the education of college students' outlook on life. Educators need to find out these problems and use appropriate means to solve them. Jiang (2020) found that in the education of college students' outlook on life, new educational ideas should be applied in combination with the times. Xu (2020) proposed that college students can be educated and guided to build a correct outlook on life in a good and positive way if the educational principles and the psychological development law of college students are followed, which will promote the healthy and positive development of college students.

Educational psychology studies the science of the basic laws of teaching and learning in the educational and teaching environment. Aubele Joseph (2021) found that educational psychology could fundamentally analyze and study the process of teaching and learning and analyze its essence, improving and optimizing the way of teaching and learning. Woerkom et al. (2021) found that positive psychology analyzes people's abilities and potential with a positive attitude. Tejada-Gallardo et al. (2021) argued that educational psychology should pay attention to people's positive emotions, positive characters, and positive educational environment.

Previous studies on educational psychology focus on the importance of college students' psychological education and positive psychology in college students' education, but there are few studies on the practical application of positive psychology to the education of college students' outlook on life. Many research conclusions provide theoretical support for the application of positive psychology in the education of college students' outlook on life, and positive psychology is a new and important basis for college students' psychological education. Based on previous studies, this paper makes innovative research on the education of college students' outlook on life under positive psychology. This study aims to obtain new ways to promote the education of college students' outlook on life through innovative research on the education of college students' outlook on life, innovate the path of college students' outlook on life, and promote educational psychology and positive psychology to play a positive role in promoting the development of the education of college students' outlook on life.

In order to study the innovation of the education of college students' outlook on life, the research background of educational psychology, positive psychology, and the education of the outlook on life are introduced. On this basis, the questionnaire is designed, and the reliability and validity of the questionnaire data are tested and analyzed by using Statistical Product and Service Solutions 25.0. Finally, the data results are further analyzed. The innovation of this paper is to combine educational psychology and positive psychology with the education of college students' outlook on life, improving the method of the education of college students' outlook on life, leading to more accurate and planned college students' outlook on life, stimulating college students' positive attitude toward life, promoting college students to form a correct and good outlook on life, and providing analysis and research data for the innovation of the education of college students' outlook on life. This study aims to provide a new way of education and research for college students' education on the outlook on life. The innovation of this paper is to mention new educational psychology which is different from traditional psychology. The theoretical research of this study is based on positive psychology. The conclusion of this study innovates the path of college students' education of the outlook on life, and makes efforts to improve the teaching quality of the college students' education of the outlook on life, making educational psychology and positive psychology play a positive role in promoting the development of the education of college students' outlook on life.

## Overview of Educational Psychology, Positive Psychology and the Education of Outlook on Life

### Overview of Educational Psychology

Educational psychology is a discipline based on psychology. It studies the learning of human beings and the effects of educational intervention, teaching psychology, and social psychology in the educational context. The focus of educational psychology is to apply the theory or psychological research to education, and the scope of the application of educational psychology is to design courses, promote students' motivation for study, and help students face setbacks in their life, learning, and growth. Educational psychology is only available in the department of education in colleges and universities (Crompton et al., 2020).

Although educational psychology is related to education and psychology, it mainly studies the psychological phenomena and laws of psychological development of students under the conditions of education and teaching, such as students' mastery of knowledge and skills, moral norms, and personality formation. Therefore, educational psychology has its characteristics. In the whole process of the study, learning and teaching is a mutual system, which includes students, teachers, teaching content, teaching environment, teaching media, and other factors, the teaching process and evaluation process interact together, having the nature of tasks of pedagogy as well as those of psychology (Hazel, 2020; I-Chia, 2020).

In the research process of educational psychology, the essential characteristics, functions, and significance of educational psychology are obtained through reviews, quantitative and qualitative methods, which is of great significance to promote the development of educational psychology (Hao, 2020).

### Overview of Positive Psychology

Positive psychology is an emerging discipline that studies traditional psychology from a positive perspective. It is a revolution in the field of psychology and a milestone in the development history of human society.

Positive psychology is a research method that absorbs most of the research methods of traditional mainstream psychology, like the scale method and questionnaire method. It combines these research methods with the phenomenological method of humanism to study happiness, advocate the positive direction of psychology, and explore the positive psychological quality of human beings, and their health, happiness, and harmonious development.

Positive psychology studies positive subjective experience, positive personality traits, and positive social environment, and analyzes the influence of positive psychology on the future development of a person's life (Jan, 2021; Tweed Roger et al., 2021).

### Overview of the Education of Outlook on Life

**Figure 1:**
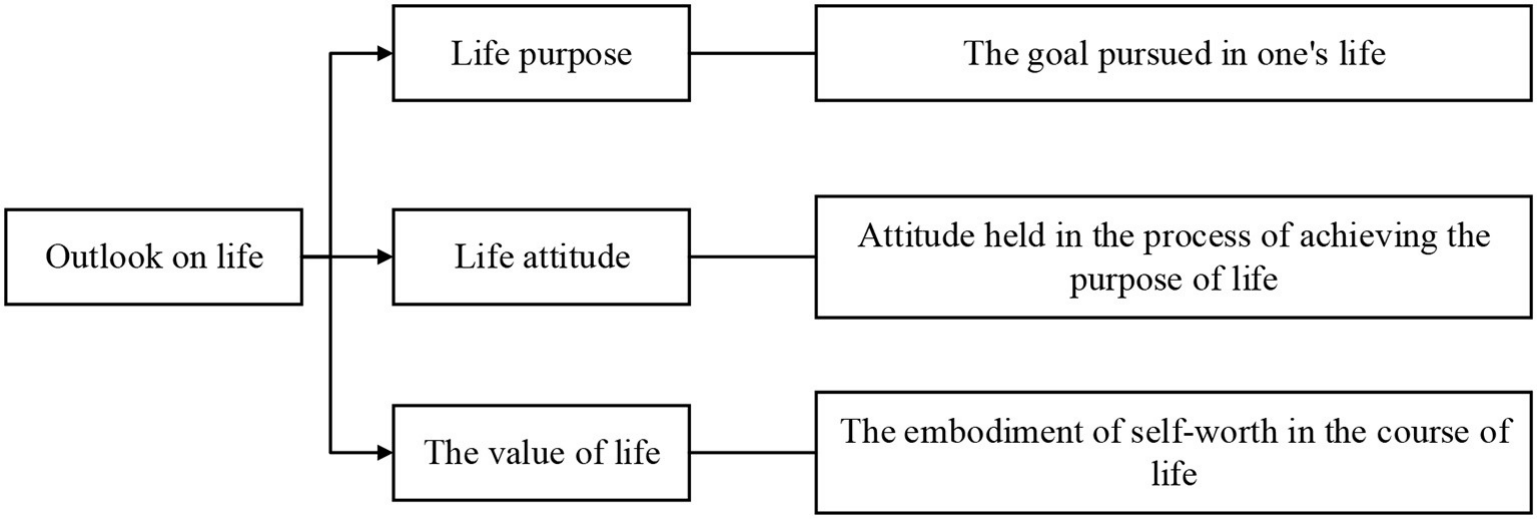
Three aspects embodied in the outlook on life.

Outlook on life is the formation of people's general and fundamental views on the purpose and significance of life, the way of life, and the way of life in the process of practice. Outlook on life determines the goal of people's life practice, guides the direction of life path, and determines the value of people's behavior and their attitudes toward life. Outlook on life is an important part of people's outlook on the world and is also restricted and influenced by the outlook on the world (Wu and Song, 2019; Prinzing Michael, 2021). In general, outlook on life is mainly reflected in three aspects, as shown in Figure 1.

Living environments and experiences have a great influence on people's outlook on life and each person's outlook on life changes with the change of the world. A correct outlook on life should be advocated and promoted because it can temper people's will, help them solve all kinds of problems and setbacks in life, and be positive in their way of life to realize self-worth (Alexiou et al., 2021).

Outlook on life can be formed through reasonable education of personal behavior, values, and attitudes toward life, which is the process of the education of outlook on life. This kind of education can realize the coordination between people's outlook on life and social development without breaking away from the mainstream. Li et al. (2019) studied the relationship between personality traits and social networking sites, and evaluated the proposed hypotheses through college students' online research. Their research results have theoretical significance in the field. For contemporary college students, the core content of ideological and political education includes the education of outlook on life. After the analysis of the basic theory of outlook on life, it is found that the education of outlook on life contains life purpose, life attitude, and the value of life. Nowadays, the connotation of the education of people's outlook on life is expressed as follows: (1) it is based on the social and human development of the modern era; (2) the goal is to pursue the overall harmonious development of life, guide people to establish the correct purpose and attitudes toward life, and realize their values. This shows that the overall harmony of life is the focus of the education of outlook on life (Qian et al., 2018; Chen et al., 2021).

The task of the education of outlook on life is to fundamentally know about the life purpose and effectively guide people to establish positive attitudes in all aspects of life. The education of outlook on life is not only ideological and political education in classrooms but also social practice and school community activities, which can reflect the self-worth of participants (Chen, 2019; Matsumoto et al., 2021).

In this study, questionnaires are used to explore the current situations of college students' outlook on life and the education of their outlook on life. The innovation of the education of college students' outlook on life is proposed based on positive psychology under the theory of educational psychology.

## Questionnaire Design

The innovation of the education of college students' outlook on life is based on positive psychology under educational psychology is studied by questionnaires. The questionnaire survey is carried out through the “questionnaire star” platform. It mainly aims at understanding and analyzing college students' outlook and the education of their outlook on life (Mathias and Peter, 2020). The senior leaders of colleges and universities were contacted to ensure the effective recovery of the questionnaire before the distribution of the questionnaires.

An expert evaluation link is also set up to evaluate the content and results of the questionnaire designed in this study, which provides some support for the effectiveness of the questionnaire.

Three colleges and universities are selected as the research object to reduce the data interference caused by the particularity of colleges and universities. In this survey, 200 questionnaires are distributed and 193 valid questionnaires are collected, with an effective rate of more than 95%. Among them, male students account for 47.3%, and females occupy 52.7%. The proportion of each grade from freshmen to seniors is balanced, and it is 27, 24.6, 23.3, and 25.1% respectively. Several key issues in the questionnaire design are shown in Table 1.

**Table 1:** Questions in the questionnaire.

**Question number**	**Questions**	**Options**
7	What do you think of life goals?	A. Pay more attention to the present life, and life goal is not necessary.
		B. When I have the goal of life, I will realize it at all costs.
		C. Setting goals for life at any stage
		D. Moving in one direction without regret
	What is the most important thing to achieve your goal in life?	A. It has to be admitted that luck matters.
		B. I rely more on a good social environment.
		C. I will be happy to accept the help of family and friends.
		D. Consider your efforts most important
	What do you choose to do when you encounter setbacks?	A. If we can't solve it, we won't think about it.
		B. Retaliation psychology
		C. I don't know what to do
		D. Believe in overcoming setbacks
17	How do you choose when your interests conflict with those of collectives and countries?	A. Sacrificing self-interest to meet collective and national interests.
		B. Priority for collective and national interests
		C. National, collective and individual interests cannot be abandoned.
		D. More emphasis on personal interests
18	What kind of life do you pursue and feel happy about?	A. Enjoying the moment and valuing self-happiness
		B. Happiness from helping others
		C. More emphasis on material life
		D. Enjoying happiness from family and work
20	How do you cultivate your values and outlook on life (Multiple choice)	A. Cultivation of parents
		B. mainly relying on their learning and exploration
		C. Values and outlook on life established under the influence of the whole society
		D. mainly the education and training of school teachers
25	What is the main influence on the education of college students' outlook on life (Multiple choice)	A. Lack of innovation in traditional educational models
		B. The content of outlook on life education is not attractive
		C. Complex information on the Internet has a great impact on me
		D. Disagree with teacher education

The above questionnaire survey shows that there are some problems in the education of college students' outlook on life. Therefore, positive psychology is introduced to try to find the solutions to the problems.

### Reliability and Validity Test of the Questionnaire

The reliability and validity, the retrograde factor analysis, and various options of the questionnaire are tested, the problem of high correlation and high overlap between variables were solved, and the multicollinearity between variables was eliminated to explore the relationship between college students' psychology and the education of their outlook on life.

The reliability analysis was carried out by SPSS 25.0. The KMO (Kaiser Meyer Olkin) value of the questionnaire data is 0.873, which is >0.8, indicating that it is suitable for factor analysis. The result of Bartlett's spherical test sig < 0.0001, which denies the original hypothesis, and the correlation coefficient matrix is a non-unit matrix. Therefore, the questionnaire data is suitable for factor analysis. This study uses exploratory factor analysis to test whether the factor load of each variable is >0.6. Through the measurement, it was found that all the factor loads in the questionnaire are >0.6, indicating that the validity of the questionnaire is good.

The reliability analysis focuses on the stability and reliability of the questionnaire. The Cronbach Coefficient α is measured by calculating the correlation coefficient of each index and combining it with the consistency test. The results show that the Cronbach coefficients α of all factors are >0.8, and the reliability of the questionnaire is good.

## Questionnaire Results and Analysis of the Education of College Students' Outlook on Life

The expert evaluation results show that the questionnaire designed in this study is effective, and the research results of this study will be described in detail. There is no significant difference in the distribution of questionnaires among the three universities in this questionnaire, so the analysis of data results takes the three universities as a research object.

### Analysis of Questionnaire Results

**Figure 2:**
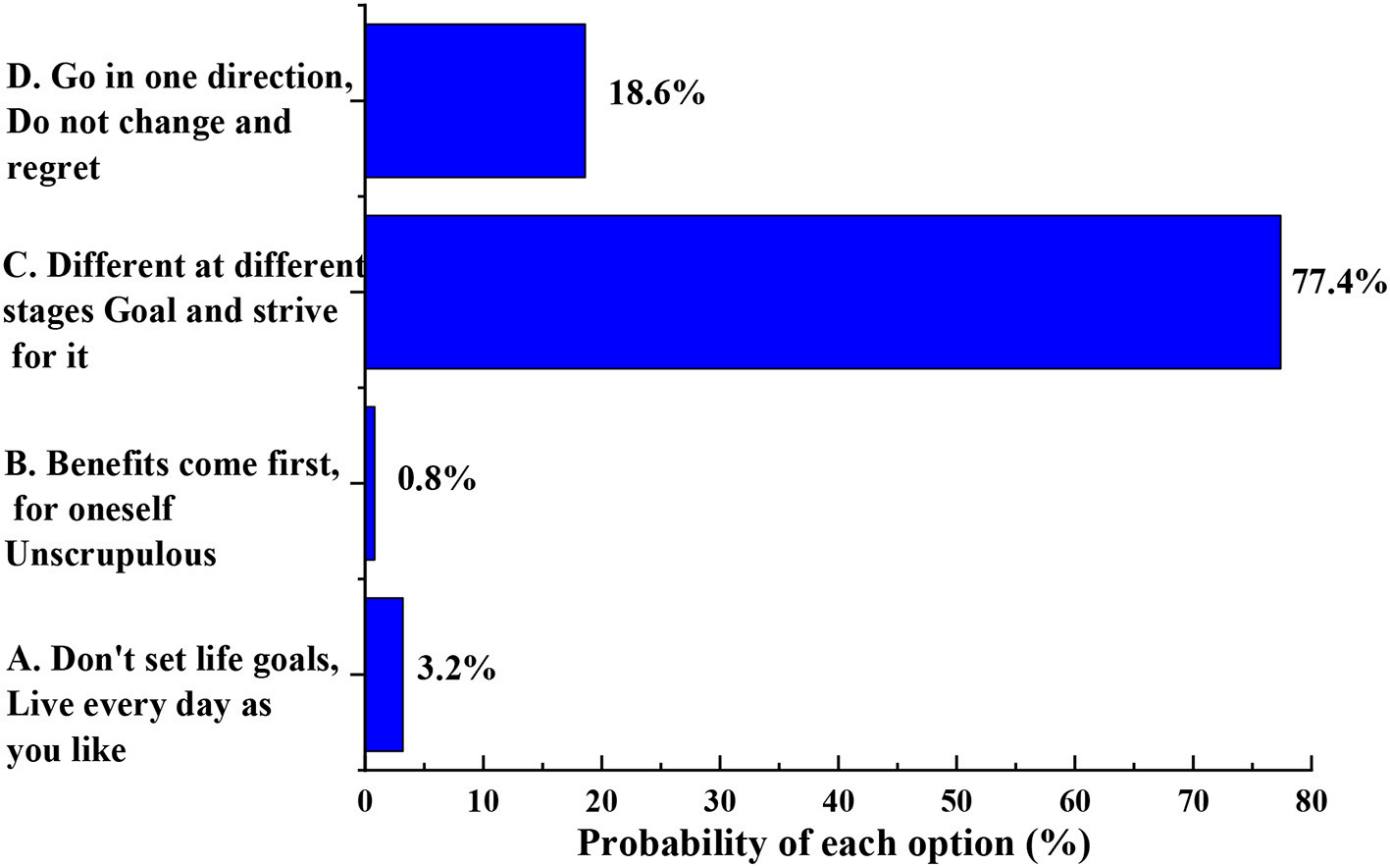
How do you view your life goals.

The problems existing in college students' outlook on life and the education of their outlook on life are shown in Figure 2.

Figure 2 shows college students' views on their life goals, which reflects college students' goals and their current situation of outlook on life. The proportion of college students who have periodical aims and work hard for them is as high as 77.4%, accounting for the largest proportion of the college students; 18.6% of college students think that they should have a goal and make it; 3.2% of college students do not set life goals and live every day at will; 0.8% of the students pay more attention to individual interests and pursue them in various ways. This shows that most college students have an accurate understanding of their life and are willing to work hard for their future life.

**Figure 3:**
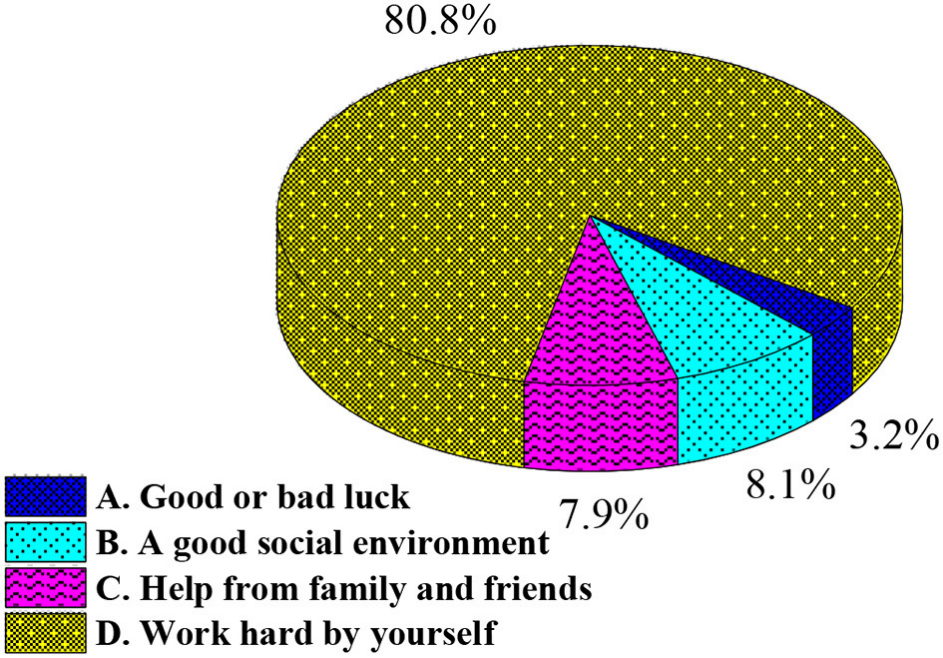
What is the most important thing that you need to achieve your life goals.

Figure 3 below shows the influencing factors affecting the setting of life goals of college students.

Figure 3 shows that the answers to question 9 in the questionnaire. This is designed according to the college students' thoughts on the influencing factors of achieving their life goals, which helps to explore the idea of college students on life goals. The results include the following: 80.8% of the students believe that the realization of life goals needs hard work, 8.1% think that life goals can be realized under a good social environment; 7.9% argue that their life goals can be realized with the help of families and friends; 3.2% believe that luck is the key to achieving life goals.

**Figure 4:**
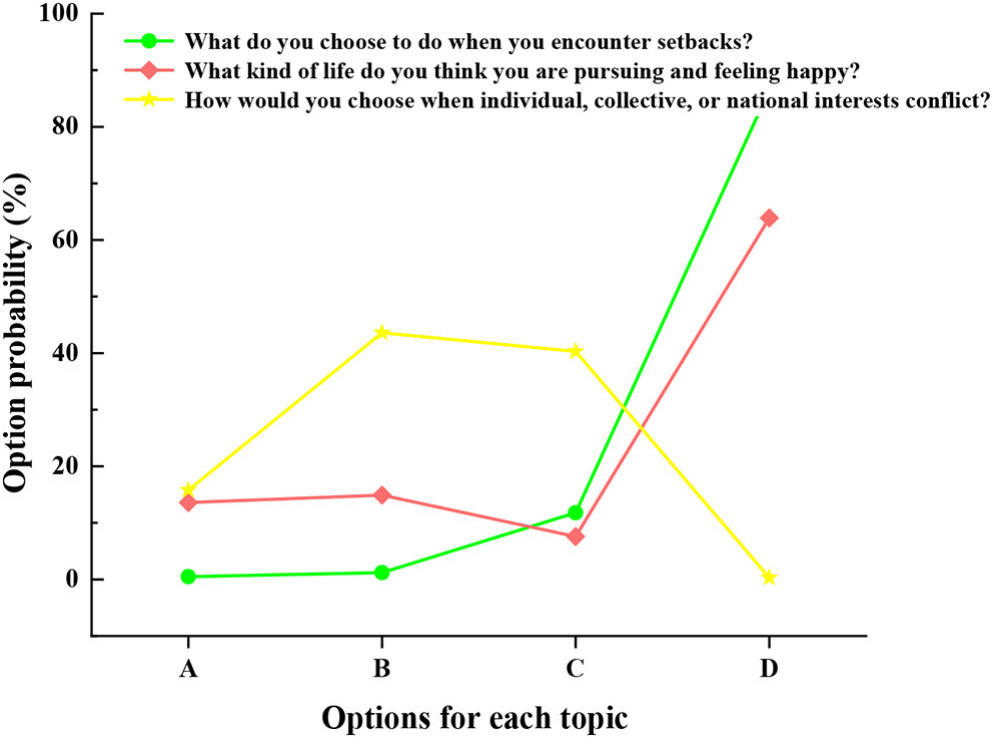
Faced with setbacks, the pursuit of happiness, and conflicts of interest, the choices made by college students.

Different students choose different answers due to the setbacks they encounter, the pursuit of happiness, and the choice of interests. Figure 4 shows the college students' options for the questions in the questionnaire.

Figure 4 shows that, in terms of the question of how to overcome the setbacks, more than 80% of college students choose to work hard, while 10% do not know how to take action; and 2% are the ones who choose to retaliate and give up when they encounter frustration. In terms of the meaning of happiness, 60% think that happiness is having a stable job and a happy family life; 18% of the students think that happiness is to help others and create self-value for society; 18% think that happiness is doing things at will; 8% think that happiness is having a lot of money and the right to speak. In terms of the question of how to choose when there is a conflict between individual, collective, or national interests: 80% of the students choose to subordinate individual interests to collective and national interests; 18% believe that they can sacrifice individual interests to protect collective and national interests; 2% choose to strive for the individual interests. Generally speaking, their choices are positive and show the current situation of college students' outlook on life and values.

**Figure 5:**
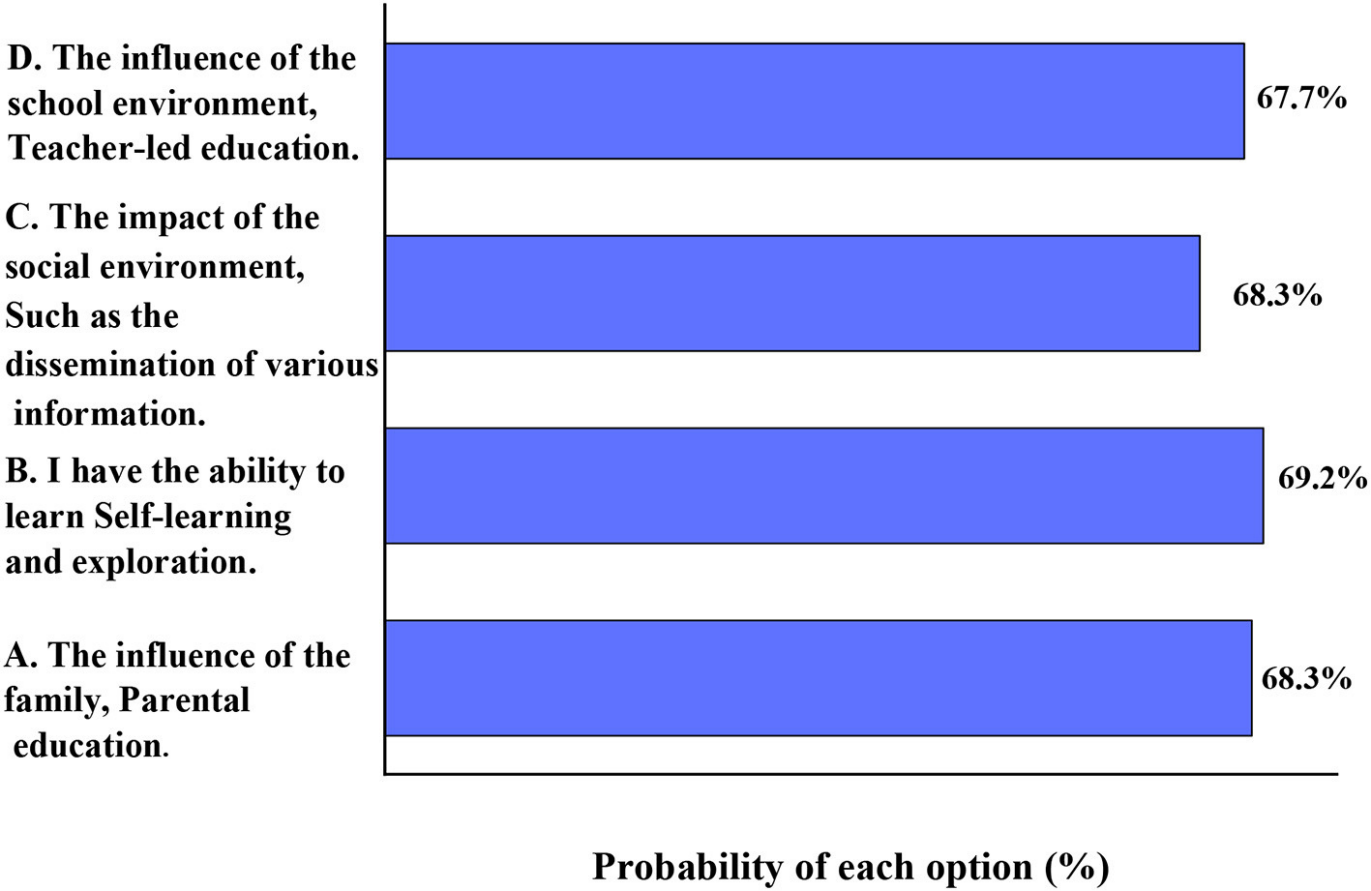
How did you cultivate your and outlook on life and values (multiple choice).

Figure 5 shows and investigated the current situation of the education of college students' outlook on life.

Figure 5 shows that the choices of the students in response to the question of the source of the education of their outlook on life: 69.2% think that the education of their outlook on life comes from self-learning and self-exploration; 68.3% think that the education of their outlook on life comes from the influence of their families and parents' education; this is also the proportion that think that social environment affects outlook on life; 67.7% believe that their outlook on life comes from school education. In general, the influence of these factors is close, that is, these factors affect the education of contemporary college students' outlook on life.

There are many problems in the education of college students' outlook on life outlook on life, and the main reason is attributed to the backward teaching methods. In the questionnaire survey, 81.8% of the college students argue that the teaching method of the education of college students' outlook on life still employs the traditional method of installation; 73.8% of the college students think that the teaching content of the education of outlook on life education is too extensive, unable to arouse their interest in the learning process, and unrelated to real life; 58% of the students think that the increasing spread of network information affects the establishment of a correct outlook on life; 62.7% think that criticism from their parents and teachers makes them lose the enthusiasm for study, thus affecting the formation of correct outlooks on life and values.

During this survey, the education of college students' outlook on life is affected by many factors, which are summarized into several important factors, as shown in Table 2.

**Table 2:** Influence factors of college students' education of the outlook on life.

**Classification**	**Influences**
Change of social environment	With the development of informatization and globalization, college students' access to information is speeding up, channels are increasing, and external influences are more.
Education models	The education model of college students should be changed according to the characteristics of contemporary college students so that college students can more easily accept the education of outlook on life.
Family education	Family is the first place to start college students' education of the outlook on life. The influence of parents and family environment will also have a subtle influence on the outlook on life.
Influence of students' ideas	Under the background of reform and opening up, the outlook on life of contemporary college students is affected by many aspects, which has a great influence on the thinking of college students and then affects the values and outlook on life of college students.

Table 2 shows that the influencing factors of the education of college students' outlook on life can be divided into four aspects. They are the influence of the social environment, the school education mode, the family education method, and students' ideological literacy. Under the influence of these factors, college students' outlook on life is formed. Correct and reliable education of outlook on life can enable college students to form a correct outlook on life (Katyaynidas, 2020).

### Analysis of Educational Psychology and the Education of College Students' Outlook on Life

There are several important aspects of educational psychology in the process of teaching and learning, as shown in Table 3.

**Table 3:** Characteristics of educational psychology.

**Characteristics**	**Influences**
Characteristics of educators	The first thing to know about educational psychology is the executors of the educational process. Their characteristics make the teaching process distinctive and have a positive impact on students' studies.
Characteristics of students	As a student, each student is different, and their reception ability is also different. Education needs to change with the characteristics of each student.
Teaching methods	Different teaching contents require different teaching methods and means. Educators need to combine educational content and methods to achieve a good teaching effect.

Table 3 shows the laws of teaching and learning from several aspects of educational psychology. When these aspects can be effectively combined, a benign educational environment and teaching methods can be formed, which is of great help to the education of college students' outlook of life.

Contemporary college students have a strong sense of autonomy, and their education of outlook on life is more rigorous and important. The current situation of the education of outlook on life has been unable to meet the needs of college students, and a more reliable and effective way needs to guide college students to establish a correct outlook on life. Rogoza et al. (2018) made an online survey of personality traits such as self-esteem, self-confidence, and narcissism, and found psychological traits play an important role in self-positioning. Many research results show that combining the content of educational psychology with the education of college students' outlook on life requires not only in-depth analysis of the characteristics of teaching and learning but also the psychology of contemporary college students. The targeted studies can greatly promote the development of college students' outlook on life and provide effective policy measures for the education of college students' outlook on life (McCrudden and Marchand, 2020; Susan, 2020).

### Analysis of Positive Psychology and the Education of College Students' Outlook on Life

**Figure 6:**
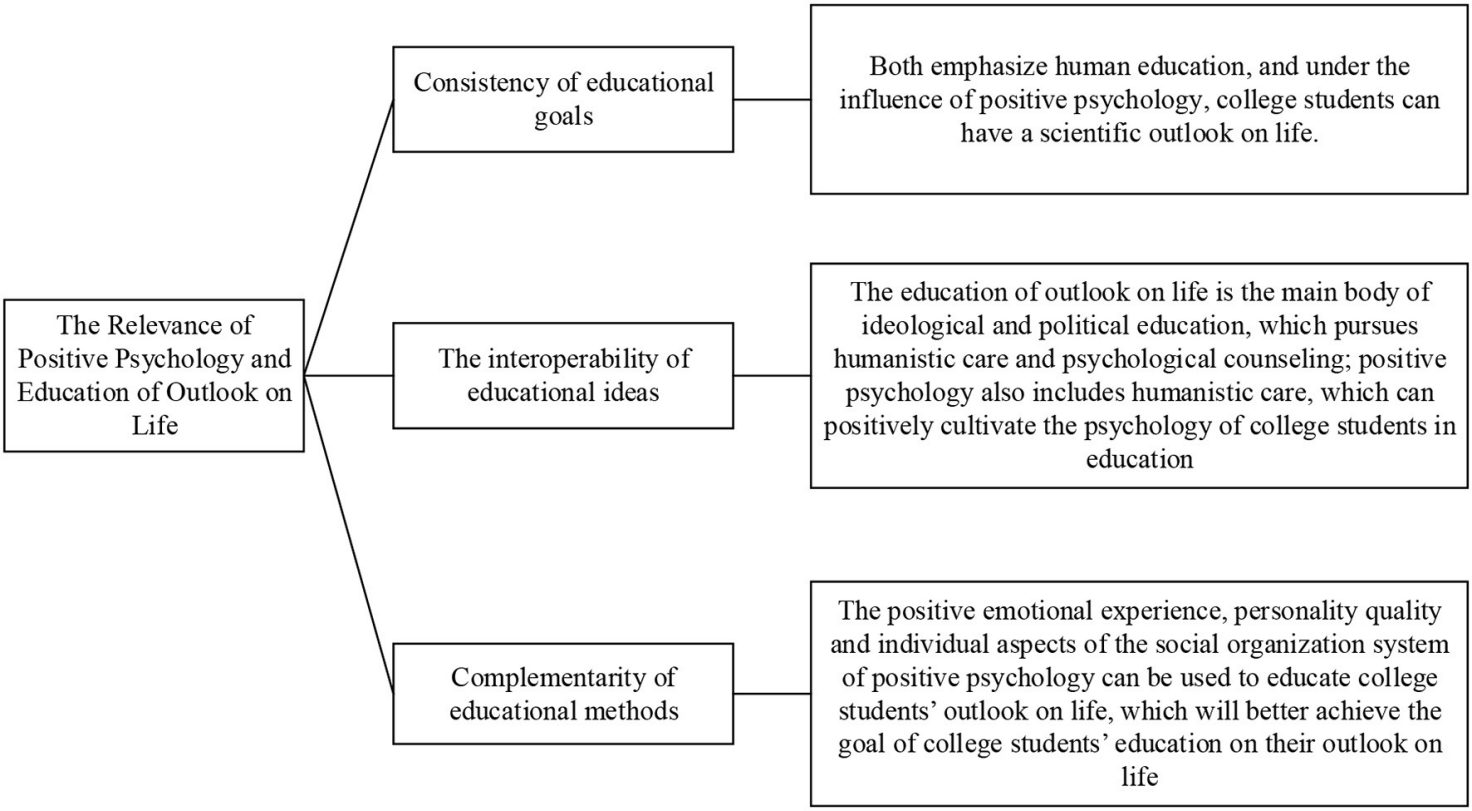
Correlation between positive psychology and the education of outlook on life.

The correlation between positive psychology and the education of outlook on life is shown in Figure 6.

Figure 6 describes the relationship between positive psychology and the education of college students' outlook on life from three aspects: the consistency of educational objectives, the interoperability of educational ideas, and the complementarity of educational methods, and it also fully illustrates the advantages of positive psychology in the education of college students' outlook on life. Its specific advantages are shown in Table 4.

**Table 4:** Advantages of positive psychology in the education of outlook on life.

**Advantages**	**Roles**
Changing the educational idea of outlook on life and advocating positive thinking	It can scientifically educate and guide college students' outlook on life, explore their positive qualities and shape their positive outlook on life
Enhancing the sense of pleasant experience and improving positive emotions	In the process of the education of College Students' outlook on life, the positive emotions of college students should be paid attention to. In a good atmosphere, college students can learn the knowledge of outlook on life with a positive attitude and have a pleasant experience
Promoting multidimensional effect and creating a positive environment	The cultivation of positive psychology for college students' positive experience and quality is combined with the surrounding environment, experiencing diversified life and activities, so that college students can realize self-worth.

Table 4 shows that the positive impact of positive psychology on the education of college students' outlook on life can be reflected by changing the concept of life education, enhancing a pleasant sense of experience, and promoting a multi-agent effect. The advantages of these three aspects can finally realize the purpose of college students' self-worth through guiding their positive outlook on life.

And positive psychology can promote the transformation of the concept of the education of college students' outlook on life, enhance the attraction of the teaching method of college students' outlook on life, and promote the actual effect of the education of college students' outlook on life. All these advantages of positive psychology can be integrated into the education of college students' outlook on life (Pagis et al., 2021).

With the rapid development of modern society, ideological and political education has become multidisciplinary and comprehensive. It absorbs nutrients from various disciplines and promotes educational methods with the times. Although positive psychology and the education of college students' outlook on life are different disciplines, the influence and effect of positive psychology on college students' outlook on life are very obvious and effective (Buijs and Jacobs, 2021).

### Innovation of the Education of College Students' Outlook on Life-Based on Positive Psychology Under the Theory of Educational Psychology

The concepts and methods of educational psychology and positive psychology are applied to the education of college students' outlook on life, and the innovative methods of the education of contemporary college students' outlook on life are put forward. This can make the education of college students' outlook on life more effective, and make the results of education more credible (Wu et al., 2019).

A positive emotional experience is needed to enable college students to establish a positive attitude toward life. First, it is necessary to cultivate college students' self-esteem and self-confidence in the education of outlook on life, which can stimulate their courage and confidence in the face of problems. Educators must strengthen students' self-education ability and expand various educational channels to make them keep confident. Second, educators can build a pleasant and relaxed classroom atmosphere by strengthening their cultivation and personality charm to improve college students' pleasant experience, so that college students have positive emotion in receiving the knowledge of the education of outlook on life. Finally, educators with the concept of happiness should popularize the correct concept of happiness to college students, so that students can establish a correct concept of happiness (Soicher et al., 2020).

College students need to have a healthy life purpose, which is achieved by cultivating college students' positive personalities. Positive psychology believes that people's sense of self is independent and active, and when college students leave their home environment for college life, they need to be independent and autonomous, making choices in the face of events. This requires the cultivation of college students' self-determination ability and lets them use their initiative. Educational psychology and positive psychology cultivate college students' optimistic spirit through scientific methods so that college students develop positive scientific habits, enhancing college students' self-confidence and living faith. Through the transfer of positive energy, contemporary college students can form a correct and positive life purpose.

The education of college students' outlook on life also needs a harmonious living environment. It is an important time for college students to form a stable sense of self-awareness and values. They are very sensitive to changes in external information. They adjust their concept of life through the exchange of information with the outside world and establish their principles and codes of conduct. Therefore, the social environment is very important at this stage. A positive social environment system is established so that this environment has a positive impact on the formation of college students' outlook on life. Also, the education system of colleges and universities should be made positive, so that students can learn the knowledge in the classroom more actively, have good interaction with teachers, and achieve a good effect on the education of outlook on life. In the whole process of the education of college students' outlook on life, their family atmosphere and strong positive psychological quality are also very important, which is conducive to the formation of college students' correct life ideal and life character, so that college students have a positive and excellent character to form a positive and correct outlook on life (Rico, 2019).

## Conclusion

As an important force for China's future development, the education of college students has always been a hot research topic. The education of outlook on life is very important for the development of college students. There are still some problems in the current education of college students' outlook on life in China. Therefore, through the analysis and research on the basic theories of educational psychology and positive psychology, the outlook on life and education of contemporary college students are analyzed, and then the application of educational psychology and positive psychology in the education of contemporary college students' outlook on life is discussed. The research results show that 77.4% of college students have periodical aims and work hard for 80.8% think that the realization of life goals relies on hard work, and this proportion accounts for the largest of the total; 80% of the students think that individual interests should be subordinate to collective and national interests when there is a conflict between individual, collective, or national interests. The proportion of students who think that the education of their outlook on life comes from self-learning and self-exploration is almost the same as that from families and parents' education, that from the social environment, and that from the school education. The above data show that college students' outlooks on life are positive. The results show that the application of the methods and theories of educational psychology and positive psychology can better grasp the thinking characteristics of contemporary college students, cultivate college students' positive attitude toward life, correct life purpose and positive life ideals, and tap the potential and ability of college students. The innovation of the education of college students' outlook on life based on positive psychology under educational psychology is put forward, and the education of college students' outlook on life needs to innovate educational environments, educational content theory, and educational methods. The combination of educational psychology, positive psychology, and the method of college students' outlook on life provides a new way to educate and study college students' outlook on life, the path of college students' outlook on life is innovated, and college students' outlook on life should be improved, in-depth study of the relevant theories of educational psychology and positive psychology and their application to teaching can play a positive role in improving college students' outlook on life. There are still some shortcomings in the study. In the questionnaire, there are a few questions about the education of college students' outlook on life, making the research results universal. In the follow-up research, more related questions should be set to deeply analyze the research problem.

## Data Availability Statement

The raw data supporting the conclusions of this article will be made available by the authors, without undue reservation.

## Ethics Statement

The studies involving human participants were reviewed and approved by East China Normal University Ethics Committee. The patients/participants provided their written informed consent to participate in this study. Written informed consent was obtained from the individual(s) for the publication of any potentially identifiable images or data included in this article.

## Author Contributions

All authors listed have made a substantial, direct, and intellectual contribution to the work and approved it for publication.

## Conflict of Interest

The authors declare that the research was conducted in the absence of any commercial or financial relationships that could be construed as a potential conflict of interest.

## Publisher's Note

All claims expressed in this article are solely those of the authors and do not necessarily represent those of their affiliated organizations, or those of the publisher, the editors and the reviewers. Any product that may be evaluated in this article, or claim that may be made by its manufacturer, is not guaranteed or endorsed by the publisher.
